# Muscle length influence on rectus femoris damage and protective effect in knee extensor eccentric exercise

**DOI:** 10.1111/sms.13890

**Published:** 2020-12-16

**Authors:** Ryoichi Ema, Kazunori Nosaka, Ryosuke Kawashima, Akihiro Kanda, Koya Ikeda, Ryota Akagi

**Affiliations:** ^1^ School of Management Shizuoka Sangyo University Iwata Japan; ^2^ School of Medical and Health Sciences Centre for Exercise and Sports Science Research Edith Cowan University Joondalup WA Australia; ^3^ College of Systems Engineering and Science Shibaura Institute of Technology Saitama Japan; ^4^ Graduate School of Engineering and Science Shibaura Institute of Technology Saitama Japan; ^5^ Mizuno Corporation Osaka Japan

**Keywords:** hip flexion, maximal voluntary contraction, muscle soreness, repeated bout effect, shear modulus

## Abstract

This study tested the hypothesis that the magnitude of rectus femoris (RF) damage and the repeated bout effect (RBE) would be greater after knee extensor eccentric exercise performed in a supine (long RF lengths) than a sitting (short RF lengths) position, and the muscle length effects would be more prominent at the proximal than distal RF. Young untrained men were placed to one of the two groups (n = 14 per group). S group performed the knee extensor eccentric exercise in the sitting position for the first bout and the supine position for the second bout, and L group performed the exercise in the supine position for two bouts, with 4 weeks between bouts. Dependent variables included evoked and maximal voluntary isometric contraction (MVC) torque, electromyography (EMG) during MVC, muscle soreness, and shear modulus, which were measured before and 1‐3 days after each exercise bout. After the first bout, L group in comparison with S group showed greater (*P* < .05) changes in hip flexor MVC torque (average of 1‐3 days post‐exercise: −11.1 ± 9.4% vs −5.0 ± 7.5%), proximal RF EMG (−22.4 ± 16% vs −9.0 ± 21.9%), and proximal RF shear modulus (33.2 ± 22.8% vs 16.9 ± 13.5%). No significant differences between groups were evident for any of other variables after the first bout including knee extensor MVC torque, and for the changes in all variables after the second bout. These results supported the hypothesis that RF damage would be greater for the spine than sitting position especially at the proximal region, but did not support the hypothesis about the RBE.

## 1 INTRODUCTION

Unaccustomed eccentric exercise induces muscle damage represented by a prolonged impairment of muscle function and delayed onset muscle soreness.^1^ The magnitude of muscle damage induced by eccentric exercise is affected by intensity, number, and velocity of eccentric contractions.^2^, ^3^, ^4^ Several studies^5^, ^6^, ^7^ demonstrated that the magnitude of muscle damage was also affected by muscle lengths during eccentric exercise. However, it appears that the study designs of the previous studies were not necessarily adequate to investigate muscle length effects on eccentric exercise‐induced muscle damage.

Nosaka et al^7^ compared an extended (from 50°‐0°, anatomical position = 0°) and a flexed (from 130°‐80°) elbow joint angle with the same range of motion (ROM) for changes in indirect markers of muscle damage after maximal eccentric exercise of the elbow flexors. They showed that the magnitude of muscle damage was greater after the exercise at the extended (longer muscle lengths) than flexed elbow joint position (shorter muscle lengths). This was also shown for the knee extensors such that greater changes in indirect markers of muscle damage were observed after knee extensor eccentric exercise performed at a flexed (70°‐110°) than an extended (30°‐70°) knee joint position.^6^ These suggest that the extent of muscle damage is greater when eccentric exercise is performed at longer muscle lengths. However, in these studies, the actual difference in the muscle length changes during eccentric exercise between the flexed and extended joint positions was not clear, since the moment arm is a function of joint angle,^8^ and neuromuscular activation^9^ and mechanical force^10^ are affected by joint angles. To clearly the effect of muscle lengths on muscle damage, it is better to compare eccentric exercises with an identical ROM in the same joint movement.

The quadriceps femoris consists of vastus lateralis (VL), vastus medialis (VM), and vastus intermedius (VI) that are monoarticular muscles crossing the knee joint, and rectus femoris (RF), which is a biarticular muscle crossing the knee and hip joints acting also as hip flexor.^11^ The proximal joint angle (ie, hip joint) affects muscle length of RF more than that of VL, VM, and VI.^8^ If muscle lengths are major determinant of muscle damage, a difference in the magnitude of changes in muscle damage markers would be observed for RF but not for vasti muscles after eccentric exercise of the knee extensors between two different hip positions (ie, flexed versus extended) with an identical knee ROM. The initial knee joint angle of the eccentric exercise in the aforementioned study by McHugh and Pasiakos^6^ was 70° versus 30°, which would correspond to ~8% difference in muscle lengths of each constituent of the quadriceps femoris between the two positions.^8^ Since RF is reported to be approximately 10% longer in supine than sitting position,^8^ the effect of muscle lengths during eccentric contractions on RF muscle damage can be better investigated by comparing a sitting and a supine position. Paschalis et al^12^ compared the changes in knee extension strength after a single bout of knee extensor eccentric exercise between sitting (shorter RF lengths) and prone (longer RF lengths) positions, but they did not isolate RF and vasti damage from the whole quadriceps femoris damage. It should be also noted that the same participants performed the knee extensor eccentric exercises in the two positions separated by 2 weeks in the study by Paschalis et al.^12^ It is possible that the results were affected by the contralateral repeated bout effect.^13^ Thus, the effects of the muscle lengths on RF damage still remain unclear.

Maeo et al^14^ investigated changes in transverse relaxation time (T2) of magnetic resonance images of RF in the proximal, middle, and distal regions, following 50 eccentric contractions of the knee extensors with the load of 90% of one repetition maximum in a sitting position. From the results of changes in T2 relaxation time and its effect size, they showed that the magnitude of muscle damage was greater at the proximal RF than other regions. However, no previous study has investigated regional differences in muscle damage in relation to RF muscle lengths. Moreover, the RF length's effect on the repeated bout effect (RBE) is not clear. Nosaka et al^7^ reported greater attenuation of changes in muscle damage markers after the second bout of eccentric exercise of the elbow flexors that was performed at the extended elbow joint angles (50°‐0°) when the first exercise bout was performed at the extended than the flexed (130°‐80°) elbow joint angle. Thus, it is possible that the magnitude of RF muscle damage is reduced greater by repeating the knee extensor eccentric exercise in the supine position than performing the exercise in the sitting position for the first exercise bout.

Therefore, the current study tested the hypothesis that the magnitude of RF damage after knee extensor eccentric exercise would be greater for the supine (long RF lengths) than sitting (short RF lengths) position, and the muscle length effect on damage would be more prominent at the proximal than distal region of RF, although damage of VL and VM would be similar between the positions. To examine the effects of RF lengths during eccentric exercise on the RBE, the present study investigated the effect of RF lengths in the initial eccentric exercise performed in supine or sitting position on muscle damage after the second eccentric exercise that was performed at the supine position. It was hypothesized that RF damage but not VL and VM damage after the second bout would be smaller when the first exercise bout was performed at the supine (long RF lengths) than sitting position (short RF lengths). Considering that RF is a key muscle for fast sprinting^15^ and RF injury is frequently occurred especially at the proximal region during eccentric contractions,^16^ to clarify RF muscle damage and its relation to muscle lengths in knee extensor eccentric exercise is important.

## 2 METHODS

### 2.1 Participants

**FIGURE 1:**
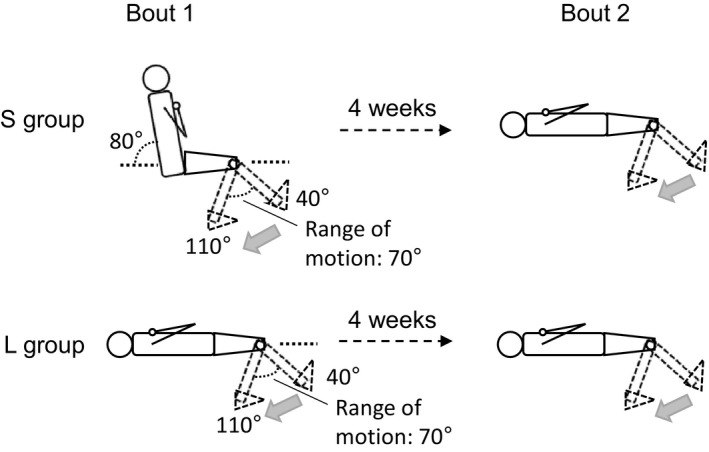
Schematic illustrations of eccentric exercise. S group completed the eccentric exercise in the sitting position for the first bout (Bout 1) and supine position for the second bout (Bout 2). L group performed the exercise in the supine positions for the both bouts. The interval between bouts was 4 wk. The angles of the hip joint and the starting and finishing knee joint angles in each exercise are shown in the illustrations of Bout 1

Healthy young men (n = 28) who had not performed habitual exercise activities at least for 6 months prior to the present study were recruited for the present study. Each participant provided a written informed consent after being informed of the study's purpose and potential risks. All participants performed two bouts of knee extensor eccentric exercise separated by 4 weeks, but the first bout exercise protocol was different between the groups (n = 14 per group). One group (age: 22 ± 1 year, height: 171.1 ± 4.1 cm, body mass: 65.9 ± 10.6 kg) completed the exercise in a sitting position (short RF lengths) for the first bout and a supine position (long RF lengths) for the second bout (S group). The other group (22 ± 1 year, 170.5 ± 7.9 cm, 63.3 ± 7.0 kg) performed the exercise in the supine position for the both bouts (L group) (Figure 1). An independent t test demonstrated that physical characteristics, physical activity during daily life assessed by International Physical Activity Questionnaire,^17^ and baseline peak torque of maximal voluntary isometric contraction (MVC) of the knee extensors at 80° hip joint and 90° knee joint angles (anatomical position = 0°) did not differ significantly between the groups.

Paschalis et al^12^ reported a large effect size (approximately 2.1 in Cohen's d) for the difference in the changes in MVC torque between the two positions in a within‐participant design. Since it was assumed to have larger variability for changes in MVC torque after knee extensor eccentric exercise among participants in a between‐participant design, the effect size of the present study was set lower. The sample size estimation (G*Power 3.1.9.4, Kiel University, Germany) showed that 12 participants per group were necessary for a medium effect size (*f* ^2^ = 0.25) with *α* = 0.05 and *β* = 0.8. Considering the potential dropouts, two more participants were recruited for each group. Throughout the experiment, the participants were requested to keep their daily activities low and not to have any interventions such as stretching and cooling of the knee extensor muscles during the experimental period. The study was approved by the Ethics Committee of the Shibaura Institute of Technology (#16‐012).

### 2.2 Eccentric exercise

Each participant sat on or lay supine on the seat of an isokinetic dynamometer (CON‐TREX MJ, PHYSIOMED, Germany) to perform eccentric exercise of the knee extensors of the right leg regardless of the leg dominance based on a preferred leg to kick a ball,^18^ while the body was secured on the seat with non‐elastic strap and seatbelt (Figure 1). The centers of rotation of the knee joint and dynamometer was carefully aligned. All participants completed two bouts of maximal eccentric exercise of the knee extensors separated by 4 weeks. Each bout of the exercise consisted of 10 sets of 10 maximal eccentric contractions with the angular velocity of 60° s^‐1^. The hip joint angle was 80° for the sitting position and 0° for the spine position (anatomical position = 0°), and ROM of the knee joint was 40° to 110° for both exercises (Figure 1). The lower thigh was passively returned to the starting position (40°) with an angular velocity of 20°·s^‐1^, with approximately 3.5 seconds interval between contractions, and 2‐minute rest was provided between sets. Verbal encouragement was provided during the exercise. According to the theoretical force‐length relations based on the cross‐bridge model for the quadriceps femoris by Herzog et al,^10^ RF was mainly on the ascending limb in the sitting position and descending limb in the supine position for the knee joint ROM used in the present study. The exercise volume during each bout was determined as the sum of 100 eccentric contraction time‐torque integrals.^5^

### 2.3 Dependent variables

Maximal voluntary isometric contraction (MVC) torque, evoked torque, electromyographic (EMG) amplitudes during MVC, muscle soreness, passive torque, and ultrasound muscle shear modulus were used as indirect markers of muscle damage.^6^, ^7^, ^19^, ^20^ They were measured just before (Pre), and every 24 hours for 3 days after each exercise bout. The measurement sites were matched among the EMG recordings, muscle soreness and shear modulus measures: at the level of 70% (VM), 50% (distal region of RF [RFd] and VL), and 30% (proximal region of RF [RFp]) of the upper thigh length which was the distance from the greater trochanter to the popliteal crease. Each dependent variable was obtained in the following order for all time points: muscle soreness, shear modulus, passive torque, evoked torque, and MVC torque with EMG.

#### 2.3.1 MVC torque

**FIGURE 2:**
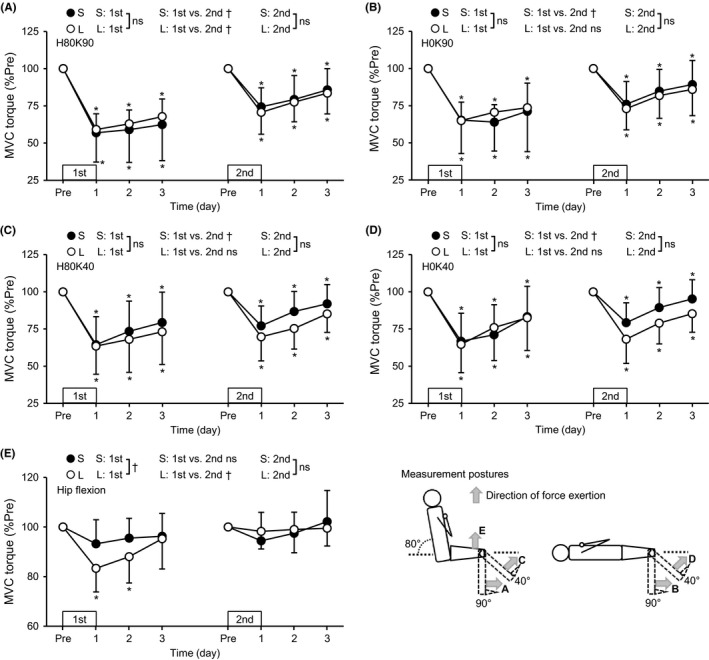
Normalized changes (mean ± SD) in peak torque of maximal voluntary isometric contraction (MVC) during knee extension at the hip joint angle of 80° and knee joint angle of 90° (H80K90 [A]), hip joint angle of 0° and knee joint angle of 90° (H0K90 [B]), hip joint angle of 80° and knee joint angle of 40° (H80K40 [C]), and hip joint angle of 0° and knee joint angle of 40° (H0K40 [D]), and during hip flexion at H80K90 (E) from the pre‐exercise value (Pre: 100%) at 1‐3 days after each eccentric exercise bout for the S and L groups. Schematic illustrations of measurement postures for the MVC torque measures are also shown in the bottom right, and each measurement of A‐E is indicated in the figure. *A significant difference from Pre. ^†^A significant interaction effect. ns: no significant interaction effect

After performing warm‐up exercise consisting of submaximal contractions at intensities of 30%, 50%, and 80% of MVC, each participant was instructed to exert isometric knee extension at two sitting and two supine positions, and hip flexion force at a sitting position (Figure 2) for 3 seconds with maximal effort twice each. For knee extension torque measures, an ankle strap was attached slightly proximal to the lateral malleolus. The knee extensor MVC torque was measured at four different combinations of the hip joint (H, 80° or 0°) and knee joint (K, 90° or 40°) angles: H80K90, H0K90, H80K40, and H0K40 (Figure 2). It has been reported that the magnitude of changes in knee extensor MVC torque after knee extensor eccentric exercise is different among the knee joint angles at which the torque is measured,^6^ but the effect of hip joint angle on the torque has not been investigated.

To examine the force produced by RF, the hip flexor MVC torque at H80K90 was also investigated (Figure 2). To assess the hip flexor MVC torque, a pad was positioned approximately 5 cm proximal from the patella (Figure 2). The participant was asked to lift the right thigh with maximal effort. The tensor fasciae latae is also a hip flexor as well as a knee extensor, but the volume of the muscle is less than one third of that of RF.^15^ Therefore, it was assumed that a change in hip flexor MVC torque represented the change in RF force generating capacity. Ema et al^11^ confirmed that the hip flexor MVC measure at H80K90 provided a good day‐to‐day repeatability (coefficient of variation = 3.6 ± 2.4%). Verbal encouragement was provided during the measurements. The means of the two contractions (MVC torque, EMG, potentiated doublet torque) were used for future analyses. Throughout the experiments, the positions of the strap and pad were consistent for each participant.

#### 2.3.2 EMG amplitudes during MVC

Surface EMG signals (Bagnoli 8 EMG System, DELSYS, USA) were obtained from the RF, VL, and VM with using pre‐amplified bipolar surface electrodes (DE‐2.1, 1 × 10 mm^2^, 10 mm inter‐electrode distance). Muscle belly and fascicle longitudinal directions were confirmed by ultrasonography (ACUSON S2000, Siemens Medical Solutions, USA). The electrodes were placed after the skin areas under the electrodes were shaved, rubbed with sandpaper, and cleaned with alcohol. The reference electrode was placed on the left medial malleolus. To match the electrode regions throughout the testing, the participants were asked to keep the marks on their skin. Torque and EMG signals were obtained at 2 kHz using an A/D converter (PowerLab16/35, ADInstruments, Australia) and stored to a computer. Analyses were conducted with LabChart software (version 8, ADInstruments, Australia), and the root mean square values of EMG signals (EMG‐RMS) of the three muscles during knee extensor MVC and RF during hip flexor MVC were determined over a 0.5‐seconds period around the maximal torque.

#### 2.3.3 Potentiated doublet torque

To measure potentiated doublet knee extension torque, supramaximal stimulus intensity by femoral nerve stimulation was determined as the intensity at which the peak twitch torque was obtained. Using a constant current variable voltage stimulator (DS7A, Digitimer Ltd, UK), a 1‐ms rectangular pulses were provided in the femoral triangle with a cathode (2 × 2 cm) and in the midway between the superior aspect of the greater trochanter and the inferior border of the iliac crest with an anode (4 × 5 cm). We did not report compound muscle action potential amplitude (M wave), because of a difficulty to detect clear wave from several participants, but no change in M wave by eccentric exercise over several days was demonstrated previously.^20^ During strength testing in H80K90 and H0K90, supramaximal doublet (100 Hz) stimulations at a higher current (≥20%) were provided 2‐second after MVC, and the potentiated doublet torque was obtained.^20^

#### 2.3.4 Passive torque

Each participant lay supine on the bench of the dynamometer, and the participant's knee joint angle was changed passively from 40° to 110° three times, at slow angular velocity of 1°/s to prevent stretch reflex. During measurement, the participants were asked so as not to exert knee extension force. We confirmed that muscle contraction was negligible based on EMG during passive movements. Passive knee extension torque was determined as the difference between the torques at 40° and 110°. The mean of the three trials were used for later analyses.

#### 2.3.5 Muscle soreness

Muscle soreness of VL, VM, and proximal and distal RF was quantified using a visual analogue scale with palpation by the investigator. The scale had a 100‐mm line with no pain on one end and extremely sore on the other end.^20^

#### 2.3.6 Muscle shear modulus

Using a B‐mode ultrasound apparatus (ACUSON S2000, Siemens Medical Solutions, USA) with a 45‐mm linear probe, longitudinal images were obtained from the resting RF, VL, and VM three times, respectively. Throughout the measurements, frequency (7 MHz), gain (0 dB), depth (8 cm), and focal point (approximately 0.5 cm below the subcutaneous adipose tissue) were unchanged. Previous studies reported that the eccentric exercise‐induced changes in resting shear modulus were dependent on muscle length at which elastography image was obtained.^19^ Therefore, the muscle length relative to optimal length was considered among the synergistic muscles. To match the region on the force‐length characteristics among the three muscles, the participants sat on a seat of the dynamometer at the hip joint angle of 40° and knee joint angle of 90°. According to a previous study,^10^ the three muscles can be on the descending limb in the sitting position. To avoid the effect of fluid shift on the obtained images, scans were taken at least 10 minutes after the participant sat on the seat.

From the longitudinal images, resting shear wave propagation speed was determined with an image analysis software (MSI Analyzer version 2.0Aql, Institute of Rehabilitation Science, Tokuyukai Medical Corporation, Japan). The shear modulus of a muscle was calculated as the product of muscle density (1084 kg/m^3^) and shear wave velocity squared.^21^ The region of interest was set as large as possible for a muscle in each image. The mean values of the three images were used in future analyses. In the measurement after the exercise bout, the ultrasonographic images were obtained while referring to the images taken at the baseline. Regarding the day‐to‐day repeatability of measurements (n = 10), the mean of the coefficient of variations was 2.5%‐5.2% for shear wave speeds.

### 2.4 Statistical analyses

A two‐way mixed‐design analysis of variance (ANOVA) with a between‐group factor (group [S, L]) and a within‐group factor (time [Pre, day 1, day 2, day 3] or bout [first, second]) was performed to compare between groups for changes in the dependent variables over time, and between bouts in each group. To examine a difference in the changes of knee extensor MVC torque among the four positions (average of 1‐3 days post‐exercise), a two‐way ANOVA (group × position) was used. When a significant main effect and/or interaction effect was evident, Bonferroni multiple comparisons were performed to examine whether the changes from baselines and group difference at each time point were significant. These statistical analyses were done by SPSS (version 25, IBM, USA). For variables that showed a significant group × time interaction effect, Cohen's d (*d*) in between‐subject designs^22^ was calculated using the average changes at 1‐3 days post‐exercise from the baseline. Based on Hopkins et al,^23^ *d* was interpreted as < 0.20, 0.20‐0.59, 0.60‐1.19, and ≥1.20 being trivial, small, moderate, and large, respectively. We considered the group difference to be substantial if *d* showed moderate or large. Significance was set at *P* < .05. Data are shown as mean ± SD.

## 3 RESULTS

### 3.1 Eccentric exercise

A significant main effect of bout (*P* = .002) without group or group × bout interaction effect was found for eccentric exercise volume. The exercise volume was greater in the second (S group: 22 259 ± 5346 Nm·s; L group: 21 275 ± 4692 Nm·s) than the first bout (S group: 19 098 ± 4405 Nm·s; L group: 19 702 ± 3468 Nm·s) without significant differences between groups.

### 3.2 MVC torque

Changes in MVC torque at different knee and hip joint angles (four for knee extensor torque and one for hip flexor torque) are shown in Figure 2. The baseline knee extensor torque at H0K40 was significantly smaller than that at H80K90 and H80K40 (*P* = .006‐.011) for the first bout, whereas no significant differences were evident between the positions for the second bout. However, the H80K40 torque for the S group and H0K40 torque for the L group were significantly greater before the second than first bout, and the H80K40 torque for the L group was smaller in the second than first bout (*P* = .022‐.045). All of the knee extensor MVC torque (H80K90, H0K90, H80K40, and H0K40) decreased at 1‐3 days post‐exercise from the baseline after the first bout (*P* ≤ .007) without significant group differences. The magnitude of the decrease was greater (*P* = .002‐.025) for H80K90 (36.7%‐40.6%) than other three positions (25.6%‐33.2%). S group showed a significant time × bout interaction effect (*P* ≤ .031) for all measures, but L group showed it only for H80K90 (*P* = .003). After the second bout, the torque decreased at all four positions from the baseline (*P* ≤ .011), and no group differences were evident. No significant differences in the magnitude of changes in all knee extensor torque after the second bout were found between groups.

For MVC torque during hip flexion, a significant group × time interaction effect (*P* = .017) was evident for the first bout. The torque decreased significantly (both, *P* < .001) from the baseline at 1 and 2 days post‐exercise only in the L group (*d* = 0.71, moderate). An interaction effect of time × bout was significant (*P* < .001) only for the L group. No significant group difference for the changes in the hip flexor MVC torque were found after the second bout.

### 3.3 EMG amplitude during MVC

**FIGURE 3:**
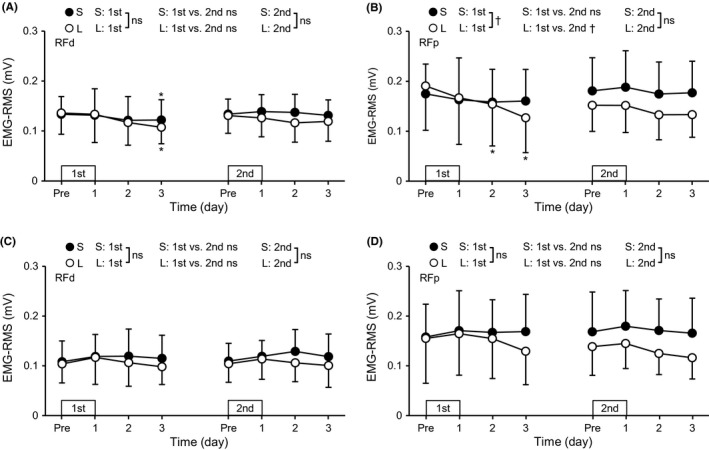
Changes (mean ± SD) in root mean square of the electromyogram (EMG‐RMS) during maximal voluntary isometric knee extension (A and B) and hip flexion (C and D) for the rectus femoris in the distal (RFd, A and C) and proximal (RFp, B and D) regions before (Pre) and 1‐3 days after each eccentric exercise bout for the S and L groups. *A significant difference from Pre. ^†^A significant interaction effect. ns, no significant interaction effect

Since no significant differences in the changes in EMG‐RMS during knee extensor MVC were found among H80K90, H0K90, H80K40, and H0K40, EMG‐RMS during knee extension MVC measures were averaged for the four positions. In the first bout, a significant (*P* = .027) group × time interaction effect was found only for EMG‐RMS of RFp during knee extension (Figure 3), and the EMG‐RMS decreased significantly (*P* ≤ .01) only for the L group (*d* = 0.70, moderate). EMG‐RMS of RFd decreased and that of VL increased by 12.8%‐13.1% in the first bout (*P* = .006‐.009) without group differences. A significant (*P* = .028) time × bout interaction effect on RFp EMG‐RMS during knee extension was only for the L group, showing smaller changes after the second than the first bout. After the second exercise bout, EMG‐RMS of VL and VM increased by 4.6%‐17.6% in both groups (*P* = .013‐.028) without significant group differences. No significant changes were evident over time for EMG‐RMS of RF during hip flexion after the first and second bouts of both S and L groups.

### 3.4 Evoked torque

**FIGURE 4:**
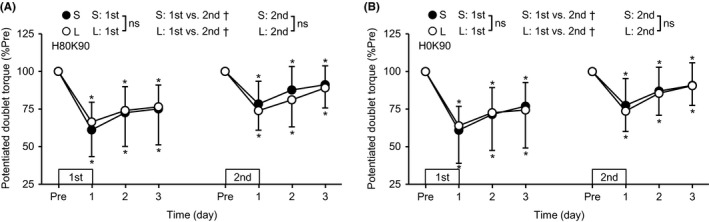
Normalized changes (mean ± SD) in potentiated double torque at the hip joint angle of 80° (A) or 0° (B) and knee joint angle of 90° (H80K90 and H0K90, respectively) from the pre‐exercise value (Pre: 100%) at 1‐3 days after each eccentric exercise bout for the S and L groups. *A significant difference from Pre. ^†^A significant interaction effect. ns, no significant interaction effect

No significant group × time interaction effect was found for the potentiated double torque in the first bout at both H80K90 and H0K90 (Figure 4). A significant main effect of time was evident for both H80K90 (*P* < .001) and H0K90 (*P* < .001), and both torque decreased by 23.1%‐39.1% from the baseline (*P* < .001). A significant time × bout interaction effect was found for the both S (H80K90, *P* < .001; H0K90, *P* = .001) and L groups (H80K90, *P* = .049; H0K90, *P* = .020), showing faster recovery in the second than the first bout for both positions. After the second bout, no significant group × time interaction effect at both positions was evident, but a significant main effect of time was found for both H80K90 (*P* < .001) and H0K90 (*P* < .001). The torque at both positions decreased by 8.9%‐26.3% (*P* ≤ .012) without significant group differences.

### 3.5 Passive torque

After the first exercise bout, the passive torque increased by 4.1%‐18.5% over three days post‐exercise without a significant difference between groups. A significant time × bout interaction effect was evident (*P* < .001) only for the L group, indicating smaller increases after the second than the first bout. The passive torque increased 2.7%‐7.3% (*P* ≤ .001) over 3 days post‐exercise after the second bout without significant group difference.

### 3.6 Muscle soreness

**FIGURE 5:**
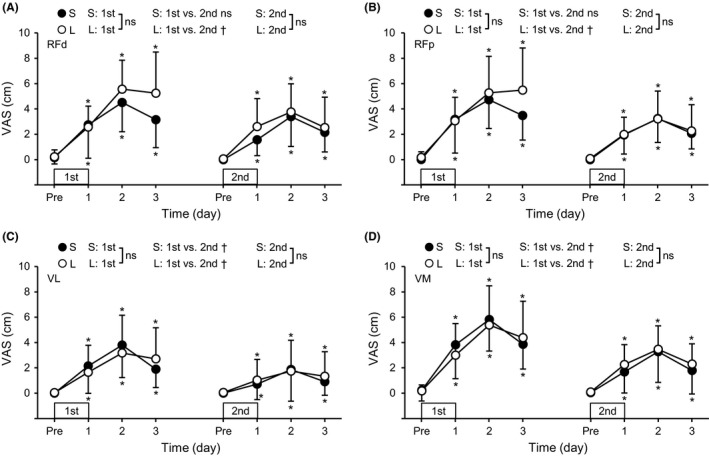
Changes (mean ± SD) in the extent of muscle soreness assessed by a visual analogue scale (VAS, a 100‐mm line) of the rectus femoris in the distal (RFd, A) and proximal (RFp, B) regions, vastus lateralis (VL, C) and vastus medialis (VM, D) before (Pre) and 1‐3 days after each eccentric exercise bout for the L and S groups. *A significant difference from Pre. ^†^A significant interaction effect. ns, no significant interaction effect

Muscle soreness developed (*P* ≤ .002) after the first bout for both groups without significant group differences (Figure 5). Except for RF in the S group, a bout × time interaction effect was significant (*P* ≤ .036), showing less muscle soreness after the second than the first bout for the three muscles in the L group and VL and VM in the S group. After the second bout, muscle soreness of all muscles increased (*P* ≤ .027), but no significant group difference was found.

### 3.7 Muscle shear modulus

**FIGURE 6:**
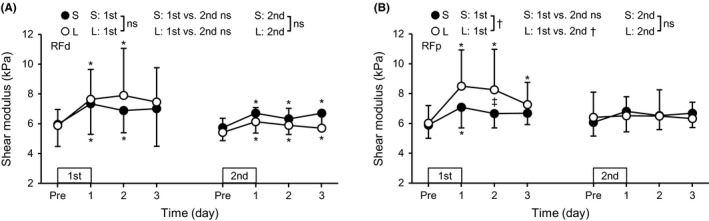
Changes (mean ± SD) in shear modulus of the rectus femoris in the distal (RFd, A) and proximal (RFp, B) regions before (Pre) and 1‐3 days after each eccentric exercise bout for the L and S groups. *A significant difference from Pre. ^†^A significant interaction effect. ^‡^A significant difference between the S and L groups. ns, no significant interaction effect

A group × time interaction effect (*P* = .044) was evident for RFp, but not for RFd, VL or VM in the first bout (Figure 6). The RFp shear modulus significantly increased at 1‐3 days post‐exercise for the L group and at 1 day post‐exercise for the S group, with greater changes in the L than S group (*d* = 1.08, moderate). The value at 2 days post‐exercise was greater (*P* = .047) for the L (8.3 ± 2.7 kPa) than S group (6.7 ± 1.0 kPa). A significant main effect of time (*P* = .001) was found for the RFd shear modulus, showing increases (*P* ≤ .008) at 1‐2 days post‐exercise after the first bout for both groups. No significant changes in the shear modulus were observed for VL and VM. When comparing bouts, an interaction effect was significant (*P* = .001) for RFp in the L group but not in the S group, showing smaller increases after the second than the first bout in the L group, and no differences between bouts were observed for RFd, VL and VM in either group. After the second bout, the modulus increased (*P* = .001‐.025) over three days post‐exercise for RFd (6.4%‐18.8%) and 1 day post‐exercise only for VM (8.6%‐10.1%) without significant group differences, whereas no significant changes were observed for RFp or VL in either group.

## 4 DISCUSSION

The hypotheses tested in the present study were as follows: 1) the magnitude of RF damage but not VL and VM damage after maximal eccentric exercise of the knee extensors would be greater for the supine (long RF lengths) than sitting (short RF lengths) position, 2) the muscle length effect on damage would be more prominent at the proximal than distal region of RF, and 3) the magnitude of RF damage but not VL and VM damage after the second bout would be smaller when the first exercise bout was performed at the supine than sitting position. After the first exercise bout, RF damage represented by changes in hip flexor MVC torque (Figure 2E), RF EMG‐RMS during knee extension MVC (Figure 3B), and post‐exercise RF shear modulus (Figure 6B) were greater for the L than S group (*d* = 0.70‐1.08), and the changes in the EMG and shear modulus were more prominent in the proximal than distal region. In contrast, changes in EMG‐RMS, muscle soreness, and shear modulus of VL and VM were not significantly different between the groups. These results appear to support the first and second hypotheses. In the second bout, no group differences were found for changes in any variables, although the changes were smaller after the second than the first bout for most of the variables, indicating the repeated bout effect (Figures 2, 3, 4, 5, 6). These results suggest no significant difference in the RBE between groups, which did not support the third hypothesis.

The present study compared changes in some indirect markers of muscle damage after eccentric exercise in the sitting versus supine position with the identical movement of the knee joint in the initial bout. It was assumed that the position difference would produce different muscle lengths during eccentric contractions for RF with similar muscle lengths for VL and VM, since it has been reported that only RF length is approximately 10% longer at the supine than sitting position.^8^ The operated region of the force‐length relationship of RF in the present study was mainly on the descending limb in the supine position and ascending limb in the sitting position.^10^ Sarcomere disruptions after eccentric exercise have been shown to occur more on the descending limb of the force‐length curve.^24^, ^25^ Thus, it was expected that greater decreases in MVC torque, evoked torque, and EMG amplitudes, and greater increases in passive torque, muscle soreness and shear modules of RF would be observed after the supine than sitting position eccentric exercise. However, the differences between the S and L groups were not clear for the four knee extension MVC torque measures (Figure 2A‐D). A significant group difference was found for the changes in the hip flexor MVC torque (Figure 2E), proximal RF EMG‐RMS (Figure 3B), and proximal RF shear modulus (Figure 6B). These results suggest that the hip flexor MVC torque, proximal RF EMG‐RMS, and proximal RF shear modulus detected the difference in RF damage better.

Muscle strength measurement is one of the best indirect muscle damage markers.^26^ Paschalis et al^12^ reported 13%‐15% differences in the decrease in the knee extensor MVC torque between sitting and prone position eccentric exercise of the knee extensors. However, the present study did not find any significant differences between the sitting and supine positions for the decreases in knee extensor MVC torque after the eccentric exercise in the different positions (Figure 2A‐D). The reasons for the discrepancy between the studies are not clear, but it is possible that the contralateral RBE might have affected the results in the study by Paschalis et al,^12^ in which the same participants were used to compare the two conditions. The RF volume is the smallest among the knee extensor quadriceps femoris, and its proportional contribution to the total quadriceps femoris is approximately 15%.^15^ Therefore, it might be that a possible effect of RF damage on the knee extension strength was masked by the large decrease in the knee extensor torque, making it difficult to detect any group difference in RF damage by comparing the changes in knee extension torque. This notion may be also the case for no group differences in the evoked knee extension torque. To the best of our knowledge, this was the first study to assess hip flexor MVC torque after knee extensor eccentric exercise. For hip flexors, Sugisaki et al^27^ showed that muscle volume of the psoas major was greater than that of other hip flexor muscles except for RF, and RF volume was greater than psoas major volume,^15^ indicating that RF is the largest muscle among the hip flexors. Accordingly, it seems likely that RF damage is detected better by the decrease in the hip flexor than knee extensor MVC torque. In fact, a significant difference in the changes in the hip flexor MVC torque was evident (Figure 2E), suggesting greater RF damage in the supine than the sitting position eccentric exercise of the knee extensors.

Decrease in EMG‐RMS during knee extension and increase in shear modulus of RF in the initial bout were greater in the L than S group, and such changes were not evident for VL and VM. This may be associated with the difference in the muscle fiber component in the RF versus VL and VM. The RF has higher percentage of type II than type I fibers when compared with other quadriceps muscles.^28^ Macgregor and Hunter^29^ showed that eccentric exercise of the knee extensors induced the reduction of the firing rate of high‐ but not low‐threshold motor units of VL, and Broos et al^30^ reported that eccentric exercise of the knee extensors increased stiffness of type IIa but not type I fibers in VL. Since type II fibers are preferentially damaged by eccentric exercise, it seems possible that RF was more vulnerable to muscle damage induced by maximal eccentric contractions, especially at long muscle lengths. The lack of changes in EMG‐RMS of RF during hip flexion is also likely to be associated with this notion. It was reported that neuromuscular activation of RF was lower during hip flexor MVC than that during knee extensor MVC.^11^ The EMG‐RMS of RF during hip flexion at H80K90 was 66%‐76% of that during knee extension at H80K90 before the first bout. This may suggest that the recruitment of high‐threshold motor units of RF was impaired greater during hip flexion than knee extension, affecting the RF EMG‐RMS during hip flexion less. In contrast, the magnitude of RF soreness was not different between the groups (Figure 5). Muscle soreness is considered to be more related to damage and inflammation of non‐contractile than contractile tissues.^1^ The inconsistency among changes in hip flexor torque, EMG, shear modulus, and muscle soreness may indicate that the muscle lengths during eccentric exercise affect muscle fibers and connective tissue surrounding muscle fibers (endomysium) and fascicles (perimysium) differently. The effects of muscle lengths on the connective tissue should be investigated further.

The group difference in RF damage represented by RF EMG‐RMS and shear modulus was more prominent in the proximal region. Motor nerve branches of RF are separated into proximal and distal regions,^31^ implying the possibility that neuromuscular control of RF during eccentric exercise is different between proximal and distal regions. Moreover, sarcomere length in vivo human muscle is not uniform between proximal and distal regions,^32^ and changes in sarcomere length during contractions are not the same between at short and long muscle lengths in an animal experiment.^33^ Thus, changes in the sarcomere lengths during the knee extensor eccentric exercise might have been different between the proximal and distal regions, and between the exercise at short versus long RF lengths. It should be also noted that proximal region of RF was the main contributor to the hip flexion torque.^34^ Therefore, greater RFp damage represented by the decrease in the EMG (Figure 3B) and increase in shear modulus (Figure 6B) seems to have resulted in the greater decrease in the hip flexor MVC torque after the eccentric exercise in the long RF lengths (L group) than the short RF lengths (S group).

It is important to note that the knee extensor MVC torque at H80K90 decreased greater (36.7%‐40.6%) than those at H80K40, H0K90, and H0K40 (25.6%‐33.2%) without group differences. This impairment could affect functional capacity, because many activities in daily living are performed with the knee and hip joint angle being flexed. McHugh and Pasiakos^6^ reported greater decreases in knee extensor MVC torque at a flexed (90° and 110°) than an extended knee joint angle (30° and 50°) in a sitting position after 120 eccentric contractions of the knee extensors, suggesting the greater strength loss at longer muscle lengths. However, this was not found in the current study in which the knee extensor MVC was measured in a supine position. Moreover, muscle lengths of each constituent of the quadriceps femoris are longest at H0K90 among the four different positions of MVC measures in the current study. Thus, the difference in the changes in the MVC torque between the flexed and extended knee joint angle may be due to the measurement posture (sitting versus supine) rather than the muscle lengths.

Regarding the RBE, no significant differences between the L and S groups were evident for the changes in the variables after the second eccentric exercise that was performed in supine position for both groups. Since the magnitude of the RBE was greater for the condition in which maximal eccentric exercise of the elbow flexors was performed at longer muscle lengths than shorter muscle lengths,^7^ it was expected to see greater protective effect for the L than S group in the present study too. It has been shown that a greater RBE is produced when muscle damage after the initial exercise bout is greater.^4^ It should be noted that the difference in the magnitude of muscle damage after the first bout between the short and the long muscle length protocols in the study by Nosaka et al^7^ was large (eg, decrease in MVC torque: 30% vs 55%, peak muscle soreness: 20.7 mm vs 31.0 mm). However, in the present study, the magnitude of the difference between the short (sitting position) and long muscle length (supine position) protocols in hip flexor MVC torque decrease was only 10%, and no significant difference between the protocols was evident for RF muscle soreness. Therefore, no significant difference in the RBE between the S and L groups in the present study was probably due to the relatively small group difference in the muscle damage or similar damage profile after the first bout. Mavropalias et al^35^ recently reported that muscle damage after the second bout of eccentric cycling in high‐intensity (repeated bout effect) was not different between the two exercise groups (low‐intensity versus high‐intensity eccentric cycling with the same volume) that showed similar changes in muscle damage markers after the initial bout. Accordingly, it seems possible that a clear difference in the RBE between protocols would have been observed when a large difference in muscle damage had been evident after the first exercise bout.

The present study had some limitations that should be considered in the future studies. Firstly, muscle damage was assessed by indirect markers only; thus, the effect of muscle lengths during eccentric exercise on the magnitude of histological changes in the muscle fibers and their surrounding structure in RF and other muscles is not known. Secondly, the present study did not observe actual RF length changes during eccentric exercise. To the best of our knowledge, RF fascicle behavior during dynamic contractions has not been investigated in previous studies, probably because of complicated fascicle arrangement in RF. For other biarticular muscles such as medial and lateral gastrocnemius, fascicle lengths in the extended knee position are longer than those in the flexed knee position,^36^ indicating that the proximal joint angle that the muscles cross affects the muscle lengths. Therefore, it is reasonable to assume that RF lengths were different between sitting and supine positions. However, it is not known how much difference in the RF length changes from the beginning to the end of each eccentric contraction and over 10 sets of 10 maximal eccentric contractions existed between the sitting and supine position eccentric exercise protocols. Thus, it should be investigated further whether initial muscle length, final muscle length, or its changes over eccentric contractions are the main factor determining the magnitude of muscle damage. Thirdly, some dependent variable measurements were taken for 3 days post‐exercise, when some of the variables did not return to the baseline yet. It is possible that the changes in some dependent variables would have been different between the groups after 3 days. Fourthly, only maximal voluntary isometric contraction torque was measured in the present study. It was reported that the magnitude of the decrease in MVC torque after knee extensor eccentric exercise was greater in eccentric than isometric contraction,^12^, ^37^ but muscle length effect was not observed in the eccentric torque.^12^ Furthermore, after an 8‐week eccentric training, both eccentric and isometric knee extension MVC torque did not change at 48 hours after the knee extensor eccentric exercise.^37^ Thus, it seems likely that the RBE was induced similarly for the eccentric and isometric torque measures, if the present study had included eccentric knee extension MVC torque measure. Lastly, the exercise regime in the present study was the single‐joint eccentric knee extensions; thus, the current findings may not be applied for multi‐joint eccentric knee extensions.

In conclusion, the current study showed that RF muscle damage was greater for the spine than sitting position. Because the effect sizes were moderate (*d* = 0.70‐1.08), the effects of RF lengths on RF damage can be interpreted as physiologically significant. In contrast, VL and VM damage was comparable between the two positions, with identical ROM during eccentric exercise. These results provide robust evidence that greater muscle damage is induced when muscle lengths during eccentric exercise are greater. Furthermore, the present study showed that muscle length effect on damage was more evident in the proximal than distal region of RF. However, the magnitude of RBE was not affected by the RF lengths during eccentric exercise of the initial bout.

## 5 PERSPECTIVE

The present study showed that RF muscle length affected the magnitude of strength loss such that the maximal knee extensor eccentric exercise in a spine position (long RF lengths) resulted in greater decreases in hip flexion torque than that in a sitting position (short RF lengths). However, the position did not appear to affect the damage of the other knee extensor muscles, and the protection against muscle damage after the subsequent eccentric exercise in the spine position. The greatest reduction of knee extensor MVC torque in H80K90 suggests that knee extension strength should be measured in a sitting position with a flexed knee joint angle. It is possible to examine RF damage after knee extensor eccentric exercise by hip flexor MVC torque measure at H80K90. To avoid severe RF damage induced by knee extensor eccentric exercise, it is better to perform the exercise in a sitting position. Histological observations after knee extensor eccentric exercise were mainly performed for VL,^1^, ^25^ and no muscle fiber damage was found.^1^ Since decreases in EMG and increases in shear modulus were more evident for RF than VL, to investigate histological changes induced by knee extensor eccentric exercise, RF should be investigated more closely.

## CONFLICT OF INTEREST

No conflicts of interest are declared by the authors.
